# Volume Change and Liver Parenchymal Signal Intensity in Gd-EOB-DTPA-Enhanced Magnetic Resonance Imaging after Portal Vein Embolization prior to Hepatectomy

**DOI:** 10.1155/2014/684754

**Published:** 2014-09-11

**Authors:** Ayako Akiba, Satoru Murata, Takahiko Mine, Shiro Onozawa, Tetsuro Sekine, Yasuo Amano, Youichi Kawano, Eiji Uchida, Shin-ichiro Kumita

**Affiliations:** ^1^Department of Radiology, Center for Advanced Medical Technology, Nippon Medical School, 1-1-5 Sendagi, Bunkyo-ku, Tokyo 113-8602, Japan; ^2^Department of Surgery for Organ Function and Biological Regulation, Nippon Medical School, 1-1-5 Sendagi, Bunkyo-ku, Tokyo 113-8602, Japan

## Abstract

*Purpose.* To investigate the liver volume change and the potential of early evaluation by contrast-enhanced magnetic resonance imaging (MRI) using gadolinium-ethoxybenzyl-diethylenetriamine pentaacetic acid (Gd-EOB-DTPA) after portal vein embolization (PVE). *Materials and Methods.* Retrospective evaluations of computed tomography (CT) volumetry of total liver and nonembolized areas were performed before and 3 weeks after PVE in 37 cases. The percentage of future liver remnant (%FLR) and the change ratio of %FLR (%FLR ratio) were calculated. Prospective evaluation of signal intensities (SIs) was performed to estimate the role of Gd-EOB-DTPA-enhanced MRI as a predictor of hypertrophy in 16 cases. The SI contrast between embolized and nonembolized areas was calculated 1 week after PVE. The change in SI contrast before and after PVE (SI ratio) was also calculated in 11 cases. *Results.* %FLR ratio significantly increased, and SI ratio significantly decreased (both *P* < 0.01). There were significant negative correlations between %FLR and SI contrast and between %FLR and SI ratio (both *P* < 0.01). *Conclusion.* Hypertrophy in the nonembolized area after PVE was indicated by CT volumetry, and measurement of SI contrast and SI ratio in Gd-EOB-DTPA-enhanced MRI early after PVE may be useful to predict the potential for hepatic hypertrophy.

## 1. Introduction

Portal vein embolization (PVE) of unilateral hepatic lobe prior to extended hepatectomy can result in liver parenchymal ischemia and induce contralateral liver hypertrophy [1–3]. This change is induced by growth factors such as hepatocyte growth factor (HGF), transforming growth factor-α (TGF-α), and epidermal growth factor (EGF) produced by the intestine and carried into the portal vein [4, 5]. They are stimulated after PVE, and hepatocyte growth is promoted as they flow into the nonembolized area preferentially [4–6]. In such cases, it is important to predict the future liver remnant (FLR) volume to perform the surgery safely. However, there is no consensus on early predictive factors based on imaging.

Gadolinium-ethoxybenzyl-diethylenetriamine pentaacetic acid (Gd-EOB-DTPA) has been used in clinical practice as a magnetic resonance (MR) contrast agent. After intravenous injection, Gd-EOB-DTPA is metabolized by 2 routes: receptor-specific uptake in hepatocytes with subsequent biliary excretion and glomerular filtration in the kidney with subsequent urinary excretion. It is eliminated in urine and feces in almost equal amounts (43.1–53.2% and 41.6–51.2%, resp.) [7, 8]. In biliary excretion, Gd-EOB-DTPA is gradually taken up by hepatocytes and eventually excreted via the biliary pathway without any change in its chemical structure [7, 8], and it has also been used for the evaluation of liver function in recent years [7, 9–14]. Based on these considerations, we assumed that change in signal intensity (SI) in Gd-EOB-DTPA-enhanced magnetic resonance imaging (MRI) could be applied in the assessment of liver parenchymal damage after PVE, and Gd-EOB-DTPA-enhanced MRI could facilitate early predictions based on imaging. The purposes of this study were to investigate the volume change in PVE prior to extended hepatectomy by computed tomography (CT) volumetry and the efficacy of Gd-EOB-DTPA-enhanced MRI early after PVE to predict potential compensated hepatic hypertrophy.

## 2. Materials and Methods

### 2.1. Patients

This study was approved by the ethics committee of our hospital. All patients provided written informed consent to receive the iodine contrast agent, Gd-EOB-DTPA, and PVE and to participate in this study. Thirty-seven patients (27 men and 10 women) with a median age of 71 years (range, 57–83) underwent PVE prior to extended hepatectomy between January 2006 and December 2013 (Table 1). Of these, 33 underwent right portal vein embolization, and 4 whose left portal veins were occluded by tumor underwent anterior segmental PVE. The underlying diseases were 18 hilar cholangiocarcinomas, 7 hepatocellular carcinomas, 5 intrahepatic bile duct cancers, 4 gallbladder cancers, 2 metastatic liver cancers, and 1 cystic duct cancer. Twenty-seven patients underwent extended right hepatectomy, and 2 patients underwent extended left hepatectomy. The other 8 patients could not undergo their predetermined operations because of a rapid increase in the size of the tumor or the emergence of lymph node metastases. Although 6 of the 7 patients with hepatocellular carcinoma had liver cirrhosis, all exhibited almost normal liver function (the Child-Pugh score was Grade A). The other 31 patients had normal livers.

All 37 patients underwent CT volumetry prior to PVE and at 21 ± 4.4 days after PVE. Additionally, prospective evaluation of Gd-EOB-DTPA-enhanced MRI was performed in 16 patients (12 men, 4 women; median age, 70.5 years; range, 57–83 years) who underwent PVE between January 2011 and December 2013.

### 2.2. PVE Technique

**Figure 1:**
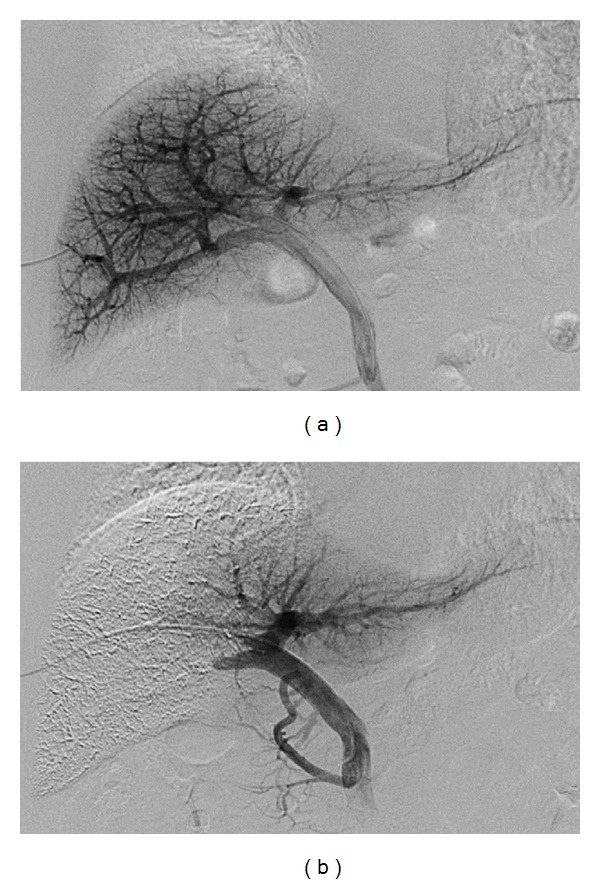
PVE procedure. (a) Direct portogram after portal vein branches were punctured via a transhepatic approach under ultrasound guidance. (b) Final portogram showing occlusion of the target portal vein branches and the patency of the veins supplying the nonembolized lobe.

All patients underwent PVE under local anesthesia approximately 3 weeks before the scheduled date of their surgery. Under ultrasound guidance, portal vein branches were punctured via a transhepatic approach with an 18 G needle (PTC needle, Hakko, Japan), and a size 5- or 6-French sheath (Super Sheath, MEDIKIT, Miyazaki, Japan) was inserted. Direct portography (Figure 1(a)) was achieved via a 4-French cobra catheter (C-MRT, MEDIKIT, Japan). Depending on the anatomy of the portal vein and the therapeutic purpose involved, the origin of the right portal vein or the anterior and/or the posterior branches were occluded with a balloon catheter (Selecon MP catheter II, TERUMO, Japan). A mixture of absolute ethanol and iodized oil (10–20 mL of absolute ethanol (maximum 0.4 mL/kg) mixed with iodized oil at a ratio of 2 : 1 ethanol : oil) was infused via a sheath or the tip of the balloon catheter. Twenty minutes after injection, embolization of the ipsilateral lobe and patency of the remnant lobe were confirmed (Figure 1(b)).

### 2.3. Retrospective Evaluation of CT Volumetry

**Figure 2:**
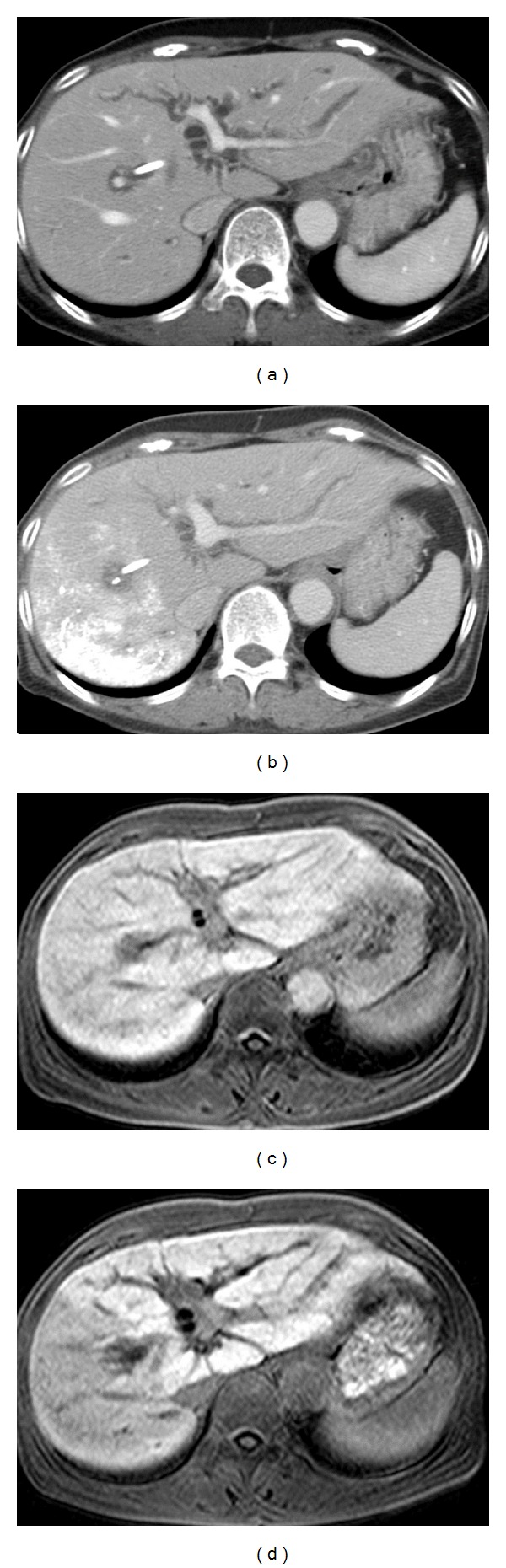
CT and MRI findings before and after PVE. (a) Contrast-enhanced CT before PVE showing a homogenous enhancement effect in the whole liver parenchyma. (b) CT 3 weeks after PVE of the right portal vein, showing accumulation of iodized oil mixed with ethanol in the right hepatic lobe and hypertrophy of the left lobe. (c) Gd-EOB-DTPA-enhanced MRI before PVE, also showing a homogenous enhancement effect. (d) Gd-EOB-DTPA-MRI 1 week after PVE of the right portal vein, showing a reduction in SI in the right hepatic lobe.

Dynamic enhanced CT was performed in all patients (*n* = 37) before and approximately 3 weeks (21 ± 4.4 days) after PVE by using mainly 64-line multidetector CT (Light Speed VCT, GE Healthcare, USA). The images in the portal phase were utilized for volumetry (Figures 2(a) and 2(b)). The areas of the total liver and the nonembolized portion were measured in each slice of 5 mm in thickness, and then total liver volume (TLV) and FLR were calculated from these data. The ratio of TLV and FLR (FLR × 100/TLV) was defined as the %FLR, and the ratio of %FLR before and after PVE (post-%FLR/pre-%FLR) was defined as the %FLR ratio.

### 2.4. Prospective Evaluation of Signal Intensity Change in Gd-EOB-DTPA-Enhanced MRI

To estimate the relationship between the degree of SI contrast after PVE and the volume change, Gd-EOB-DTPA-enhanced MRI was performed approximately 1 week after PVE (7.31 ± 1.2 days) in 16 patients. Of these, 11 patients also underwent MRI before PVE. In all cases, a 1.5 T MRI system (Intera, Philips, The Netherlands) was used. The parameters used were 3D GRE, TR 3.36 ms, TE 1.08 ms, and a flip angle of 15°. The images were obtained 15 minutes after the injection of Gd-EOB-DTPA (Figures 2(c) and 2(d)).

Average SIs in liver parenchyma without vessel and bile duct in the embolized and nonembolized areas were measured in 3 slices from each area. The ratio of the average SIs in the embolized area and the nonembolized area (SI in embolized area/SI in nonembolized area) was defined as SI contrast. Furthermore, the ratio of the SI contrast before and after PVE (post-SI contrast/pre-SI contrast) was defined as the SI ratio.

### 2.5. Statistical Analysis

All data were analyzed with the Statistical Package for the Social Sciences software (version 20, SPSS, Chicago, IL). The changes in %FLR and SI contrast were analyzed with Student's *t*-test. The correlations between %FLR ratio and both post-SI contrast and SI ratio were analyzed via Spearman's correlation. Differences with a significance value of *P* < 0.05 were considered statistically significant.

## 3. Results

The PVE procedure was successfully performed in all patients. There were 28 patients with fever of more than 37.5°C, and all fevers subsided within 5 days. Eleven patients required nonsteroidal anti-inflammatory drugs for abdominal pain. None of the patients showed pulmonary complications such as pulmonary hypertension and embolism on clinical examination (less than Grade 1 in Common Terminology Criteria for Adverse Events version 4.0). Other major complications that could potentially impede the operation did not occur. Twenty-nine patients could undergo the scheduled operation. Although the other 8 patients could not undergo their predetermined operations, this was not due to any adverse effect of PVE but due to acute progression of cancer.

### 3.1. Changes in %FLR

**Figure 3:**
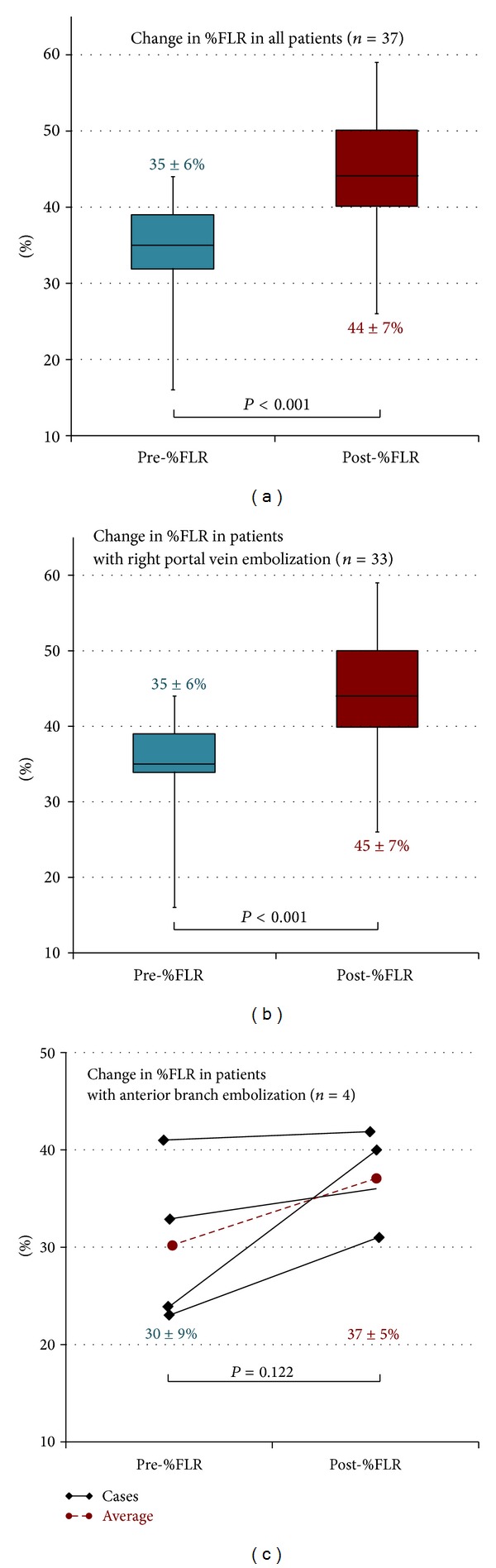
Changes in %FLR. (a) %FLR increased significantly after PVE in all 37 cases (*P* < 0.001). (b) In 33 cases in which the right portal vein branch was embolized, %FLR increased significantly after PVE (*P* < 0.001). (c) In 4 cases in which the anterior segmental portal vein was embolized, there was not a statistically significant difference in %FLR before and after PVE (*P* = 0.122).

The mean pre-%FLR and post-%FLR values were 35% ± 6% and 44% ± 7%, respectively. %FLR increased significantly after PVE (*P* < 0.001, Figure 3(a)). In the patients whose right portal vein branches were embolized, the mean pre-%FLR was 35% ± 6% and the mean post-%FLR was 45% ± 7%. %FLR increased significantly after PVE (*P* < 0.001, Figure 3(b)). In patients whose anterior segmental portal veins were embolized, the mean pre-%FLR was 30% ± 9% and the mean post-%FLR was 37% ± 5%. There was no significant difference in %FLR before and after PVE (*P* = 0.122, Figure 3(c)).

### 3.2. Pre-SI Contrast and Post-SI Contrast

**Figure 4:**
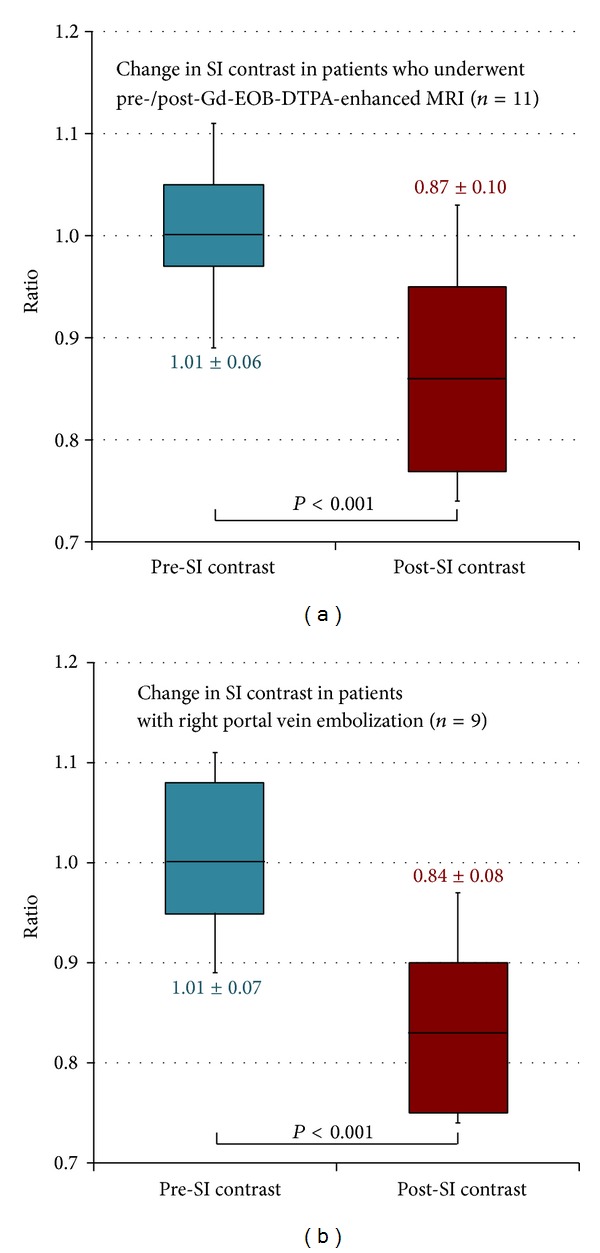
Changes in SI contrast. (a) SI contrast decreased significantly after PVE (*P* < 0.001) in 11 patients who underwent Gd-EOB-DTPA-enhanced MRI before and after PVE. (b) In 9 patients whose right portal vein branches were embolized, SI contrast decreased significantly (*P* < 0.001).

The mean pre-SI contrast was 1.01 ± 0.06, and the mean post-SI contrast was 0.87 ± 0.10. SI contrast decreased significantly after PVE (*P* < 0.001, Figure 4(a)). In the patients whose right portal vein branches were embolized, the mean pre-SI contrast was 1.01 ± 0.07 and the mean post-SI contrast was 0.84 ± 0.08. SI contrast decreased significantly (*P* < 0.001, Figure 4(b)). In the cases of anterior segmental portal vein embolization, the mean pre-SI contrast was 1.02 ± 0.00 and the mean post-SI contrast was 1.01 ± 0.04. There was not a significant difference in SI contrast before and after PVE (*P* = 0.66).

### 3.3. %FLR, Post-SI Contrast, and SI Ratio

**Figure 5:**
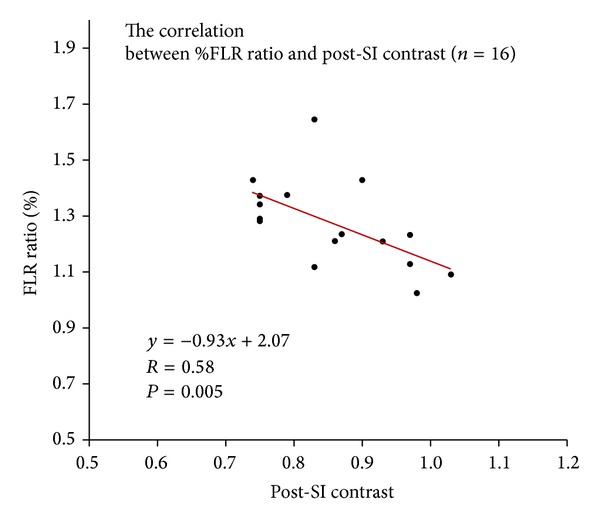
The correlations between %FLR ratio and post-SI contrast. There was a significant negative correlation between %FLR ratio and post-SI contrast (*P* = 0.005) in 16 patients who underwent Gd-EOB-DTPA-enhanced MRI after PVE.

There were significant negative correlations between %FLR ratio and post-SI contrast (*P* = 0.005, Figure 5) and between %FLR ratio and SI ratio (*P* = 0.001). In the patients whose right portal vein branches were embolized, the difference in the negative correlation between %FLR ratio and post-SI contrast was not statistically significant (*P* = 0.065). There was a significant negative correlation between %FLR ratio and SI ratio (*P* = 0.007). In the cases of anterior segmental portal vein embolization, the correlations could not be informatively analyzed because the number of cases was not large enough.

## 4. Discussion

Generally, the degree of hepatocyte proliferation is directly proportional to the degree of stimulation [15]. Surgical resection or trauma can encourage the rapid growth of viable hepatocytes in the remnant liver, and liver function can normalize within 2 weeks, even where the damage extends to up to two-thirds of the liver [16–18]. On the other hand, the peak in the generation of hepatocytes after PVE is delayed 3-4 days in comparison with that after liver resection [6]; apoptosis after PVE is thought to be weaker than that after liver resection. Thus, it takes approximately 2–6 weeks to achieve sufficient hypertrophy of the residual parenchyma [1]. It is important to accurately predict potential compensated hepatic hypertrophy early after PVE to facilitate safe surgery.

To evaluate the damage to hepatocytes after PVE, we prospectively measured the SI change in Gd-EOB-DTPA-enhanced MRI based on the following rationale. The SI of liver parenchyma after injection of Gd-EOB-DTPA depends on its uptake by hepatocytes and biliary excretion [7, 9–14]. Uptake of Gd-EOB-DTPA shortens the T1 relaxation time of the liver [9–14]. It is also known that liver damage compromises the transporter that incorporates Gd-EOB-DTPA [10, 14]. Therefore, insufficiency of hepatocytes impedes the uptake of Gd-EOB-DTPA and decreases SI [10]. In this study, post-SI contrast was significantly reduced in comparison with pre-SI contrast. This suggests that PVE results in inhibition of the incorporation of Gd-EOB-DTPA in embolized areas. Thus, the reduction in SI contrast may reflect the degree of liver damage.

A significant negative correlation between %FLR and SI ratio was observed in this study; lower SI contrast was associated with higher %FLR. Thus, it could be considered that the FLR volume will increase more if the damage to the embolized area and the reduction in uptake of Gd-EOB-DTPA are more severe. On the basis of the principle of hepatocyte regeneration, the uptake of Gd-EOB-DTPA in the nonembolized area might increase. Hence, if the difference in the degree of contrast enhancement by Gd-EOB-DTPA between the embolized and nonembolized areas is greater, the FLR volume may be predicted to increase more. The same result was apparent in the patients whose right portal vein branches were embolized.

On the other hand, in the patients whose anterior segmental portal veins were embolized, %FLR did not increase as in the patients whose right portal vein branches were embolized. Furthermore, the mean SI contrast hardly changed (from 1.02 to 1.01), in contrast to the significant decrease in the mean from 1.01 to 0.84 evident in the cases of right portal vein embolization. We surmise that the reason is as follows. Occlusion of left portal venous branches by tumor invasion or oppression had occurred gradually in the course of tumor progression at the time of PVE. In this process, a gradual influx of the growth factors into the total right lobe had already commenced; hence, the degree of stimulation of hepatocyte generation in the posterior lobe after PVE of the anterior segmental branch only was not sufficient, being less than that of the patients who underwent PVE of the whole right portal vein.

In this study, although the patients whose SI contrast decreased significantly in Gd-EOB-DTPA-enhanced MRI achieved acceptable hypertrophy, the patients whose SI contrast changed only slightly did not achieve sufficient hypertrophy. This suggests that hypertrophy of nonembolized area is less likely to be achieved in cases with negative SI change 1 week after PVE. At that time, switching to another treatment instead of surgery or additional procedures such as arterial embolization can be considered. This decision can be made earlier than in cases followed only by CT performed 3 weeks after PVE. Thus, Gd-EOB-DTPA-enhanced MRI soon after PVE may provide useful information with regard to selecting an adequate therapeutic alternative, even in the patients with rapidly growing tumors.

Our study has 2 main limitations. We utilized the ratio of embolized and nonembolized areas as an indicator of the uptake of Gd-EOB-DTPA, and these data were considered adequate for prediction of the degree of liver volume change after PVE. However, some studies have used absolute values corresponding to T1 relaxation times via the look-locker sequence for the evaluation of liver function [19–21], and we are considering further evaluation using these parameters for the early evaluation of PVE. Second, our sample size was not large, which limited the capacity for analysis of all factors affecting %FLR and SI contrast. Particularly, differences in the parameters of hepatocytes between the right and left hepatic lobes would be more informatively identified with a larger number of patients; we are planning a subsequent study focusing on this aspect.

In conclusion, the tendency for hypertrophy to develop in the nonembolized area after PVE of the right portal branch was indicated by CT volumetry. Moreover, the SI contrast measurement between the embolized and nonembolized areas in Gd-EOB-DTPA-enhanced MRI 1 week after PVE may be a feasible predictor of the potential for hepatic hypertrophy.

## Figures and Tables

**Table 1:** Patient characteristics (*n* = 37).

Parameters	Patients, *n* = 37
Sex (men/women)	27/10
Age (median [range])	71 (57–83)
Underlying disease	
Hilar cholangiocarcinoma	18
Hepatocellular carcinoma	7
Intrahepatic bile duct cancer	5
Gallbladder cancer	4
Metastatic liver cancer	2
Cystic duct cancer	1
Operative procedure	
Extended right hepatectomy	27
Extended left hepatectomy	2
None∗	8
Embolized vessel	
Right portal vein	33
Anterior segmental branch	4

*Operation could not be performed owing to rapid increase in tumor size and so forth.
